# Factors Associated with Cigarette Smoking Cessation in Lao People’s Democratic Republic: Results from the 2015 National Adult Tobacco Survey

**DOI:** 10.3390/ijerph17144953

**Published:** 2020-07-09

**Authors:** Thanh Cong Bui, Phonepadith Xangsayarath, Daovieng Douangvichith, Latsamy Siengsounthone, Khatthanaphone Phandouangsy, Ly Thi-Hai Tran, Michael S. Businelle

**Affiliations:** 1 Department of Family and Preventive Medicine, Stephenson Cancer Center, Oklahoma Tobacco Research Center, The University of Oklahoma Health Sciences Center, Oklahoma City, OK 73104, USA; Michael-Businelle@ouhsc.edu; 2 National Center for Laboratory and Epidemiology, Ministry of Health of Lao PDR, Vientiane, Laos; phonepadithxangsayarath@gmail.com; 3 Ministry of Health of Lao PDR, Vientiane, Laos; ddaovieng2@gmail.com; 4 Lao Tropical and Public Health Institute, Ministry of Health of Lao PDR, Vientiane, Laos; slatsamy@yahoo.com; 5 Secretariat of the National Tobacco Control Committee, Department of Hygiene and Health Promotion, Ministry of Health of Lao PDR, Vientiane, Laos; tphandouangsy@yahoo.com; 6 Oklahoma Medical Research Foundation, Oklahoma City, OK 73104, USA; Ly-Tran@omrf.org

**Keywords:** tobacco use, cigarette smoking, cessation, Lao People’s Democratic Republic

## Abstract

Cigarette smoking represents a major public health problem in Lao People’s Democratic Republic (Lao PDR). This study aims to examine factors associated with cigarette smoking cessation attempts and intention to quit. Data were from the Lao National Adult Tobacco Survey that consisted of 7562 participants ≥15 years old. Multivariable logistic regression models were used to evaluate the associations, adjusted for sex, age groups, education level, income per day, and smoking frequency. Results show that past quit attempts were associated with visiting a healthcare provider in the past year (adjusted odds ratio [AOR]: 1.74, 95% confidence intervals [CI]: 1.28–2.35), home smoking bans (AOR: 5.52, 95% CI: 2.13–14.33), noticing media-based messages informing the dangers of smoking or encouraging quitting (AOR: 3.25, 95% CI: 2.28–4.63), noticing health warnings on cigarette packages in the past 30 days (AOR: 3.33, 95% CI: 2.21–5.03), and believing that smoking is seriously harmful to their health (AOR: 3.45, 95% CI: 1.24–9.57). The Lao PDR government should continue implementing tobacco control policies that demonstrated associations with cessation attempts or intention to quit, such as smoke-free environments and required health warnings on cigarette packages. Tobacco cessation treatment programs are pressingly needed in Lao PDR.

## 1. Introduction

Cigarette smoking represents a major public health problem in developing countries [1,2] and in Lao People’s Democratic Republic (Lao PDR) specifically. In our previous report of the 2015 Lao National Adult Tobacco Survey (NATS), the prevalence of cigarette smoking was 48.9% in men and 5.3% in women [3]. Current tobacco use was more prevalent among groups with older ages and groups with lower education levels.

When the NATS 2015 data were collected, Lao PDR had several national policies and strategies for tobacco control, following the World Health Organization’s MPOWER measures (Monitoring tobacco use and tobacco control policies; Protecting people from the dangers of tobacco smoke [i.e., smoke-free policies]; Offering help to quit tobacco [i.e., cessation programs]; Warning the public about the dangers of tobacco, including health warnings on cigarette packages and anti-tobacco mass media campaigns; Enforcing bans on advertising, promotion and sponsorship; and Raising tobacco taxes) [4,5,6]. Specifically, smoke-free environments were enforced in many public facilities in Lao PDR, including healthcare facilities, educational facilities, and government-owned offices and workplaces [4,7]. Tobacco taxes were levied, though total tobacco prices after taxes in Lao PDR were still lower than those in other Southeast Asian countries [4,8,9]. Tobacco advertising was comprehensively banned, and health warning images and messages on cigarette packages were required. The National Tobacco Control Committee (NTCC) of Lao PDR coordinated with several ministries to implement and enforce all of these measures. Despite these achievements, Lao PDR lacked some critical programs for tobacco control. Specifically, there was no large-scale tobacco treatment program such as a national toll-free telephone quitline [4]. Although a few small-scale pilot telephone or in-person counseling programs were tested, funding limitations prevented large-scale or nationwide implementation. Nicotine replacement therapy and other medications to treat tobacco dependence, most of which were unofficially imported, were available over-the-counter at some retail pharmacies; however, they were available only in large cities and were not covered by national health insurance.

Using NATS 2015 data, this study aims to examine individual-level factors that were associated with successful cigarette smoking cessation, making a quit attempt, and intention to quit in the future. Findings of this study will provide useful information to reinforce or modify tobacco control strategies in Lao PDR and other countries in the region.

## 2. Materials and Methods

The Lao NATS 2015 used the Global Adult Tobacco Survey (GATS) standard protocol [10], including sampling strategies, selected age range, and measures. Briefly, the Lao NATS consisted of a nationally representative sample of 7562 participants ≥15 years old recruited nationwide through a stratified 2-stage cluster sampling approach. Provinces served as strata (*n* = 18), and villages or comparable urban administrative units served as primary sampling units (PSU) in each stratum. At the first stage, PSUs were selected by using the probability proportional to size method. At the second stage, 20 households were selected from each PSU through a circular systematic sampling method with a randomly selected starting household (total sample: 2969 households). All eligible people ≥15 years old in selected households were invited to participate (participation rate: 85%, with an average of 2.5 persons/household participated). In Lao PDR, people ≥15 years old can give consent and are expected to complete compulsory education, which lasts 9 years from age 6 to age 14. The survey included questions about demographic characteristics, various tobacco use practices, smoking characteristics, awareness of harms caused by tobacco, exposure to secondhand smoke, and beliefs related to tobacco use (e.g., smoking causes illnesses or cigarettes’ price should be increased). The MPOWER measures were used as a framework for the survey questions as well as for our analyses. The questionnaires were administered by CommCare software on tablets. All interviewers were thoroughly trained to conduct the survey to ensure standardization. Further details of the Lao NATS 2015 can be found in our previous publication [3]. The study received ethical review and approval from the Lao National Ethics Committee for Health Research (IRB00006227).

The main dependent variables in this analysis were (i) being a former cigarette smoker (i.e., already quit), (ii) current smokers who made a quit attempt in the past year (i.e., past quit attempts), and (iii) current smokers who did not make a quit attempt in the past year but planned to quit in the future (i.e., intention to quit). Current cigarette smokers were participants who answered “daily” or “less than daily” to the question, “Do you currently smoke tobacco daily, less than daily, or not at all?” and answered ≥1 to a question about the number of cigarettes smoked per week. Former cigarette smokers were those who did not currently smoke but answered “daily” or “less than daily” to the question, “In the past, have you smoked tobacco on a daily basis, less than daily, or not at all?” and answered ≥1 to a question about the number of cigarettes that participants smoked per week in the past. Making a quit attempt in the past year was defined as using any methods to try to stop smoking during the past 12 months, including counseling, pharmacotherapy, traditional medicine, and self-quitting (cold turkey). Based on the question, “Which of the following best describes your current thinking about quitting smoking?” current smokers who responded, “planning to quit within the next month,” “thinking about quitting within the next 12 months,” or “I will quit someday but not within the next 12 months,” were considered planning to quit in the future; while those who responded, “I am not interested in quitting,” were considered not planning to quit. Unless specified, responses/values of almost all independent variables or covariates (e.g., except age groups) listed in the tables are in their original forms of measures.

We used Stata 14.2 (StataCorp, College Station, TX, USA) to perform statistical analyses and accounted for the complex sampling design and sampling weights. We used appropriate tests (e.g., chi-square, binary logistic regression) to examine bivariate associations between dependent variables and sociodemographic and behavioral characteristics of interest. We used different multivariable logistic regression models to evaluate associations between each dependent and independent variable, adjusted for sex, age groups, education level, income per day, and smoking frequency. All *p*-values were two-tailed and were considered statistically significant if <0.05.

## 3. Results

Table 1 displays the sociodemographic and behavioral characteristics of current and former smokers in Lao PDR. Most current smokers were male (89%), lived in rural areas (77%), smoked daily (92%), and more than half of smokers (61%) had an average income below the poverty line. Most current smokers were married (87%) and were allowed to smoke at home (97%). Sixty three percent of former smokers and 45% of current smokers agreed that the price of cigarettes should be raised to encourage cessation.

Former smokers accounted for 19% of all ever smokers (Table 2). Among current smokers, 29% made a quit attempt in the past year. Common methods or aids used to quit included unaided smoking cessation (~90% for both former and current smokers), counseling (5% for former smokers and 9% for current smokers), switching to tobacco chewing (8% in former smokers and 1% in current smokers), and nicotine replacement therapy or other prescribed medications (1% in former smokers and 3% in current smokers). Among current smokers who did not make a quit attempt in the past year, 11% planned to quit in the future.

Compared with current smokers, former smokers were more likely to visit a healthcare provider in the past year (adjusted odds ratio [AOR]: 1.86, 95% confidence interval [CI]: 1.23–2.80), less likely to be exposed to secondhand smoking at indoor workplaces in the past 30 days (AOR: 0.33, 95% CI: 0.15–0.71), more likely to have a smoking ban in place at home (AOR: 4.86, 95% CI: 3.42–6.92), and more likely to believe that smoking causes illnesses (AOR: 1.79, 95% CI: 1.04–3.08). Among current smokers, past quit attempts were associated with visiting a healthcare provider in the past year (AOR: 1.74, 95% CI: 1.28–2.35), more likely to have a smoking ban in place at home (AOR: 5.52, 95% CI: 2.13–14.33), more likely to report noticing media-based messages informing the dangers of smoking or encouraging quitting (AOR: 3.25, 95% CI: 2.28–4.63), more likely to report noticing health warnings on cigarette packages in the past 30 days (AOR: 3.33, 95% CI: 2.21–5.03), and more likely to believe that smoking is seriously harmful to their health (AOR: 3.45, 95% CI: 1.24–9.57). Among current smokers who did not make a quit attempt in last year, intention to quit in the future was associated with noticing media-based messages informing the dangers of smoking or encouraging quitting (AOR: 2.83, 95% CI: 1.34–5.96), noticing health warnings on cigarette packages (AOR: 8.31, 95% CI: 2.88–23.96), belief that smoking causes illnesses (AOR: 5.06, 95% CI: 1.29–14.97), and non-belief that smoking made a real man (AOR: 0.34, 95% CI: 0.12–0.96). There were no significant interactions among examined independent variables.

## 4. Discussion

Our study found several individual-level factors that were associated with being a former vs. current smoker or intention to quit, including the beliefs that smoking is harmful or causes illnesses, visiting a healthcare provider in the past year, no exposure to secondhand smoking at workplaces, having a smoking ban in place at home, and noticing media-based messages informing the dangers of smoking or encouraging quitting. Of current smokers, 65% noticed media-based messages informing the dangers of smoking or encouraging quitting (almost all of these media-based messages were implemented by NTCC), 71% noticed health warnings on cigarette packages in the past 30 days, and between 63–73% believed that smoking is seriously harmful to their health or causes illnesses. These proportions are comparable to those in neighboring countries; for example, 67% of current smokers in Vietnam and Thailand reported thinking about quitting because of health warning labels on cigarette packages [11]. In this study, these factors were associated with approximately three times greater odds of making a quit attempt in the previous year. These results suggest that the relevant tobacco control strategies in Lao PDR (e.g., media campaigns and required health warnings on cigarette packages) have contributed to raising smokers’ awareness of the harms of tobacco smoking and may have motivated them to quit. Similar effects of these tobacco control strategies on cessation attempts or intention have also been observed in other member countries that implement the MPOWER measures [11,12,13]. Specifically, an analysis of GATS data from several developing countries, including Indonesia, Malaysia, Thailand, and Vietnam, showed that large pictorial warnings were associated with a lower cigarette smoking prevalence, particularly among those who had a lower level of education [13]. It is estimated that, among the MPOWER measures, health warnings contribute to preventing the largest number of smoking-attributable deaths (13.3 million, compared to <1 million for each of the other MPOWER policies) [14]. Therefore, the Lao NTCC should continue these efforts. 

Visiting a healthcare provider in the past year was associated with making quit attempts and quitting smoking. Those Lao smokers who sought healthcare might have had a tobacco-related disease, might have been advised to quit, or might have been more aware of the benefits of quitting. Another plausible explanation was that seeking healthcare and quitting smoking had no causal relationship and might be independent effects of a common cause such as individuals’ care for their health. Regardless of the plausibility, this result suggests that a visit to a healthcare provider offers a teachable moment for smoking cessation treatment in Lao PDR, especially when the cause of the visit is related to tobacco use. Unfortunately, most of the current smokers in this study (80%) reported never receiving advice to quit from a healthcare provider. Although simple advice to quit from a healthcare provider can prompt a cessation attempt, a systematic review showed that simple advice to quit often had a small effect [15]. Another systematic review showed that hospital-based smoking cessation behavioral interventions are effective when supportive contacts continue for at least 1 month [16]. These may explain the lack of an association between cessation intention and ever receiving advice to quit from healthcare providers in this study. In the United States, healthcare providers are recommended to ask patients about tobacco use, advise all identified smokers to quit, and assist them to quit or connect them to a smoking cessation program, such as the 5As approach (Ask, Advise, Assess, Assist, and Arrange) [17] or Ask-Advice-Connect approach [18,19]. These evidence-based approaches have been shown to be effective in increasing cessation attempts and successful cessation. Given the fact that most large healthcare facilities in Lao PDR are public and under the Ministry of Health’s supervision, the Lao government may consider establishing a system that motivates healthcare providers to ask patients about their smoking status at each medical visit, advise smokers to quit, and enroll them in an evidence-based smoking cessation program.

Similar to previous studies on this topic and as expected in the MPOWER policy package [6], exposure to secondhand smoking at indoor workplaces was associated with lesser likelihood to quit smoking. Two-third of smokers in Lao PDR reported exposure to secondhand smoking at indoor workplaces; this proportion is higher than that in Thailand (27%) and in Vietnam (56%), and is comparable to that in China (63%) [11]. Exposure to co-workers who smoked might reinforce the peer and subjective norms that favor smoking behavior or might trigger craving in those who are trying to quit. Thus, maintaining and expanding national policies regarding smoke-free working environments may facilitate cessation [6]. Similarly, there is evidence in other countries that the implementation of smoke-free homes increased intention to quit and quitting activity [20]. In this study, most current smokers were married and almost all could smoke at home (97%). Indeed, 87% of Lao people reported exposure to secondhand smoke at home, making home the most common place of secondhand smoke exposure in Lao PDR [9]. This prevalence was also the highest in Southeast Asian countries, compared to 78% in Indonesia (the second highest) and 22% in Brunei (the lowest) [9]. Thus, national campaigns in Lao PDR should discourage smoking indoors at hometo facilitate cessation in smokers and to protect their families from secondhand smoke. 

Several studies in Asian countries that implemented the MPOWER policy package have found that a significant proportion of tobacco users want to quit, yet cessation support services and facilities are often lacking [21,22]. Lao PDR is facing a similar situation. In this study, nearly one-fifth of ever smokers have quit (i.e., became former smokers) and nearly one-third of current smokers made quit attempts in the past year. Despite these encouraging cessation numbers, it is important to note that approximately 90% of smokers who tried to quit, did so cold turkey without medications or counseling. There is a pressing need for effective, affordable, and widely accessible tobacco cessation treatment programs in Lao PDR to reduce smoking and smoking-related morbidity and mortality.

Although 63% of former smokers and 45% of current smokers agreed that the price of cigarettes should be raised to encourage cessation, smokers’ income was not associated with cessation or intention to quit, cautioning policy making. In our previous analysis, approximately 12% of smokers’ family incomes were spent on cigarettes [3]. Other studies in low- and middle-income countries, such as Cambodia, have also found that low income or poverty did not appear to decrease the use of tobacco [23,24] or increase cessation [25]. Higher price (i.e., tax) of cigarettes has been widely advocated to prevent smoking initiation or reduce smoking in smokers [26,27]. Some other evidence, however, suggest that cigarette tax increase has a small effect in reducing cigarette smoking, particularly among moderate or heavy smokers [28,29]. For heavy smokers, demand for cigarettes or nicotine is more inelastic. As aforementioned, effective and affordable tobacco treatment programs are not currently publically available in Lao PDR [4,7], and the cost of over-the-counter nicotine replacement therapy is equal to or greater than cigarettes and is paid fully out-of-pocket. In the absence of low-cost and effective tobacco treatment services, increases in tobacco taxes may result in a greater proportion of smokers’ low household incomes being used to purchase cigarettes. Therefore, it is critical to have affordable and effective tobacco treatment programs in Lao PDR prior to further increases in tobacco taxes.

This study has some limitations. Because the survey is cross-sectional, directions of the relationships are uncertain. In addition, the small unweighted numbers of participants in some subgroups resulted in wide confidence intervals and potentially limited the power to detect associations. Responses to several questions about tobacco-related beliefs were binary (i.e., yes/no) instead of on a Likert-scale, and hence may not accurately reflect nuance degrees in participants’ perceptions or opinions.

## 5. Conclusions

Tobacco control policies and campaigns in Lao PDR have contributed to raising smokers’ awareness of the harms caused by tobacco smoking and may have increased intentions to quit. Nearly one-third of current smokers attempted to quit in the past year and another 11% planned to quit in the future. There is a pressing need for effective, affordable, and widely accessible tobacco cessation treatment programs in Lao PDR to help these smokers attain their goal of becoming smoke-free. Also, the Lao PDR government should continue tobacco control policies that demonstrated an association with cessation or intention to quit, such as smoke-free environments and required health warnings on cigarette packages, in order to facilitate smoking cessation and prevent smoking-related morbidity and mortality.

## Figures and Tables

**Table 1:** Demographic characteristics of adult cigarette smokers, Lao People’s Democratic Republic, 2015, weighted % (95% CI).

Characteristics	Former Smokers (Unweighted *N* = 484)	Current Smokers (Unweighted *N* = 1941)	Current Smokers Who Made a Quit Attempt in the Past Year (Unweighted *N* = 519)	Current Smokers Who Planned to Quit in the Future (Unweighted *N* = 138)
Sex				
Male	76.5 (66.1–86.9)	89.3 (86.4–92.2)	93.3 (89.8–96.9)	89.5 (84.8–94.2)
Female	23.5 (13.1–33.9)	10.7 (7.8–13.6)	6.7 (3.1–10.2)	10.5 (5.8–15.2)
Age group (years)				
15–24	4.4 (2.6–6.3)	7.8 (6.2–9.4)	8.8 (5.1–12.5)	9.2 (5.3–13.2)
25–34	12.0 (9.3–14.8)	16.3 (14.3–18.4)	18.2 (14.7–21.6)	17.5 (11.3–23.6)
35–44	21.6 (16.2–27.0)	24.3 (22.6–26.0)	24.9 (19.4–30.3)	33.3 (27.7–39.0)
45–54	26.5 (23.2–29.8)	24.4 (22.3–26.5)	24.3 (18.3–30.5)	20.5 (12.4–28.5)
≥55	35.5 (29.7–41.3)	27.2 (24.7–29.6)	23.8 (19.3–28.3)	19.5 (13.5–25.6)
Residence				
Urban	30.0 (−1.8–61.9)	23.2 (3.1–43.4)	32.6 (0.9–64.3)	22.8 (1.4–44.1)
Rural	70.0 (38.1–101.8)	76.8 (56.6–96.9)	67.4 (35.7–99.1)	77.2 (55.9–98.6)
Ethnicity				
Lao	49.9 (31.0–68.9)	58.2 (46.9–69.6)	58.9 (41.7–76.0)	65.0 (46.6–83.5)
Others ^a^	50.1 (31.1–69.0)	41.8 (30.4–53.1)	41.1 (24.0–58.3)	35.0 (16.5–53.4)
Religion				
None	4.3 (0.9–7.8)	2.2 (0.9–3.5)	1.1 (0.0–2.2)	3.1 (–1.2–7.4)
Buddhist	65.0 (48.0–82.0)	71.2 (61.3–81.2)	75.8 (63.9–87.7)	77.3 (63.0–91.6)
Others ^b^	30.7 (15.5–45.9)	26.6 (17.0–36.2)	23.1 (11.4–34.8)	19.6 (8.1–31.0)
Marital status				
Never married	4.1(1.3–7.1)	7.7 (5.9–9.5)	9.0 (6.2–11.8)	12.2 (7.3–17.2)
Currently married	89.5 (86.6–92.4)	87.0 (84.8–89.3)	85.7 (80.6–90.8)	85.0 (80.9–89.1)
Divorced/Separated/Widowed	6.4 (5.0–7.8)	5.3 (4.2–6.4)	5.3 (2.4–8.2)	2.8 (0.5–5.0)
Education level				
Never attended school	23.1 (13.4–32.8)	16.8 (13.6–20.0)	8.5 (5.6–11.5)	14.0 (4.2–23.7)
Primary school	41.1 (32.8–49.5)	46.0 (40.7–51.4)	42.3 (31.5–53.1)	40.9 (32.7–49.1)
Secondary school	18.7 (14.2–23.2)	23.5 (21.4–25.7)	27.0 (23.1–31.0)	27.3 (19.9–34.9)
High school or higher	17.1 (5.3–29.0)	13.7 (7.6–19.7)	22.2 (9.2–35.1)	17.8 (8.7–26.8)
Average income per household member per day in US dollars				
<1.9 ^c^	56.3 (41.2–71.3)	61.3 (54.2–68.3)	55.5 (45.1–65.9)	57.9 (41.9–73.9)
≥1.9	43.7 (28.7–58.8)	38.8 (31.7–45.8)	44.5 (34.1–55.0)	42.1 (26.2–58.1)
Occupation				
Unemployed	10.4 (6.0–14.9)	9.0 (7.1–11.0)	7.1 (4.1–10.2)	3.6 (0.0–7.2)
Government sector	9.3 (4.0–14.5)	6.7 (4.5–9.0)	9.8 (6.4–13.3)	9.7 (5.7–13.7)
Non-government company/organization	17.1 (12.7–21.4)	14.3 (10.9–17.7)	10.4 (4.5–16.3)	6.3 (–0.2–12.8)
Agriculture	50.9 (36.1–65.6)	58.9 (51.0–66.7)	58.5 (43.3–73.8)	64.0 (48.4–79.7)
Non-farm self-employed	6.0 (3.2–8.9)	7.0 (4.1–9.9)	9.7 (5.9–13.5)	12.1 (3.4–20.8)
Others	6.4 (3.9–8.8)	4.1 (2.8–5.3)	4.5 (1.8–7.2)	4.3 (1.6–7.0)
Smoking frequency				
Daily	80.1 (74.0–86.2)	92.3 (91.1–93.5)	85.2 (82.4–88.0)	89.6 (83.8–95.4)
Occasional (less than daily)	19.9 (13.9–26.0)	7.7 (6.5–8.9)	14.8 (12.0–17.6)	10.4 (4.6–16.2)
Number of cigarettes smoked per day, median (Q1–Q3)		10 (6–20)	10 (5–16)	10 (5–15)
Time to smoking the first cigarette of the day after waking				
≤30 min	36.0 (25.1–46.8)	40.4 (36.0–44.8)	39.6 (32.8–46.4)	38.9 (27.0–50.7)
>30 min	64.0 (53.2–74.9)	59.6 (55.2–64.0)	60.4 (53.6–67.2)	61.1 (49.3–73.0)
Methods used to quit				
In-person or telephone counseling/ support	5.2 (2.1–12.2)		8.5 (5.4–13.2)	
Nicotine replacement therapy or other prescribed medications	1.3 (0.2–8.2)		3.2 (2.1–5.0)	
Traditional medicine	1.5 (0.2–8.7)		1.4 (0.7–2.9)	
Switching from smoking to chewing tobacco, including betel quid	8.4 (3.8–17.7)		0.9 (0.4–2.2)	
Unaided cessation (cold turkey)	91.0 (76.8–96.9)		89.1 (85.1–92.1)	
Visited a health-care provider during the past 12 months				
No	63.9 (56.6–71.2)	80.5 (78.4–82.6)	73.4 (68.5–78.4)	84.2 (78.5–90.0)
Yes	36.1 (28.8–43.5)	19.5 (17.4–21.6)	26.6 (21.6–31.5)	15.8 (10.0–21.5)
Ever received advice to quit smoking from health care providers				
No	75.9 (61.0–90.7)	80.5 (76.0–85.1)	75.5 (69.9–81.0)	86.4 (72.1–100.8)
Yes	24.1 (9.3–39.0)	19.5 (15.0–24.0)	24.5 (19.0–30.1)	13.6 (–0.8–27.9)
Exposed to secondhand smoking at indoor workplace in the past 30 days				
No	33.0 (21.5–44.6)	16.9 (9.8–24.0)	20.5 (12.1–28.9)	40.8 (24.7–56.9)
Yes	67.0 (55.4–78.5)	83.1 (76.0–90.2)	79.5 (71.1–87.9)	59.2 (43.1–75.3)
Exposed to secondhand smoking at indoor public places (including buildings, healthcare facilities, restaurants, food store, and public transportation vehicles) in the past 30 days				
No	62.3 (55.5–69.2)	64.9 (58.3–71.6)	63.0 (58.7–67.3)	56.0 (44.6–67.5)
Yes	37.7 (30.8–44.5)	35.1 (28.5–41.7)	37.0 (32.7–41.3)	44.0 (32.5–55.4)
Smoking rule at home				
Allowed	86.8 (76.4–97.2)	97.4 (95.1–99.9)	94.1 (89.5–98.6)	100.0
Not allowed	13.2 (2.8–23.6)	2.6 (0.2–5.0)	5.9 (1.4–10.5)	0
Noticed media-based messages informing the dangers of smoking or encouraging quitting in the past 30 days				
No	32.7 (25.4–40.0)	35.0 (30.7–39.3)	16.8 (12.8–20.7)	23.1 (10.6–35.6)
Yes	67.3 (60.0–74.6)	65.0 (60.7–69.3)	83.2 (79.3–87.2)	76.9 (64.4–89.4)
Noticed health warnings on cigarette packages in the past 30 days				
No		29.0 (25.8–32.3)	16.4 (11.9–20.8)	6.7 (2.6–10.9)
Yes		71.0 (67.7–74.2)	83.6 (79.2–88.1)	93.3 (89.1–97.4)
Believed that smoking is harmful to participants’ health				
No, not harmful	6.6 (4.2–10.3)	3.5 (2.8–4.5)	1.9 (0.5–0.5)	2.3 (0.5–5.1)
Yes, a little harmful or moderately harmful	20.1 (15.4–25.7)	34.0 (30.3–38.0)	19.7 (12.7–12.7)	23.6 (8.0–39.3)
Yes, seriously harmful	73.3 (65.9–79.5)	62.5 (58.5–66.2)	78.4 (71.2–71.2)	74.1 (58.6–89.5)
Believed that smoking causes illnesses (including bronchitis, lung cancer, or heart diseases)				
No	16.3 (10.4–24.7)	27.0 (20.7–34.4)	22.1 (15.4–15.4)	7.4 (1.3–16.1)
Yes	83.7 (75.3–89.6)	73.0 (65.7–79.3)	77.9 (71.2–71.2)	92.6 (83.9–101.3)
Believed that it is a sin for a cigarette or pipe smoker to produce smoke that is inhaled by other persons.				
No	12.8 (8.8–18.3)	14.0 (11.8–16.6)	13.9 (11.1–11.1)	7.9 (3.5–12.3)
Yes	87.2 (81.7–91.2)	86.0 (83.4–88.2)	86.1 (83.3–83.3)	92.1 (87.7–96.5)
Believed that “A man who does not smoke is not a real man”				
No	89.1 (86.8–91.1)	84.9 (81.3–87.9)	84.4 (79.7–79.7)	92.5 (86.9–98.1)
Yes	10.9 (8.9–13.2)	15.1 (12.1–18.7)	15.6 (10.8–10.8)	7.5 (1.9–13.1)
Believed that the price of cigarettes should be raised to encourage people to stop smoking				
No	37.5 (29.7–46.1)	55.3 (51.8–58.8)	45.5 (40.5–40.5)	43.3 (36.0–50.6)
Yes	62.5 (53.9–70.4)	44.7 (41.2–48.2)	54.5 (49.5–49.5)	56.7 (49.4–64.0)

^a^ Including PhouThai, Khermou, Khamu, Khmu, Leu, Mong, etc. ^b^ Including Christian, Pee, Phi, Phy, Pi, etc. ^c^ International poverty line (http://povertydata.worldbank.org/poverty/country/LAO).

**Table 2:** Factors associated with smoking cessation and cessation intention among adult cigarette smokers, Lao People’s Democratic Republic, 2015.

Characteristics	Former Smokers (Unweighted *N* = 484) vs. Current Smokers (Unweighted *N* = 1941) (Total Sample: Ever Smoked Cigarettes, *N* = 2425)			Current Smokers Who Made a Quit Attempt in the Past Year (Unweighted *N* = 519) vs. Who Did not (Unweighted *N* = 1281) (Total Sample: Current Smokers Who Answered the Question about Past Quit Attempts, *N* = 1800)			Current Smokers Who Planned to Quit in the Future (Unweighted *N* = 138) vs. Those Who Did not (Unweighted *N* = 1141) (Total Sample: Current Smokers Who Did not Make a Quit Attempt Last Year, *N* = 1279)		
	Weighted % of Former Smoker (95% CI)	Unadjusted OR (95 % CI)	Adjusted OR ^d^ (95 % CI)	Weighted % of Those Who Made A Quit Attempt Last Year (95% CI)	Unadjusted OR (95 % CI)	Adjusted OR ^d^ (95 % CI)	Weighted % of Those Who Planned to Quit (95% CI)	Unadjusted OR (95 % CI)	Adjusted OR ^d^ (95 % CI)
Total	19.2 (15.4–23.8)			28.6 (23.7–34.0)			10.6 (7.9–13.9)		
Sex									
Male	17.0 (12.9–21.9)	1	1	29.9 (24.6–35.8)	1	1	10.8 (8.1–14.3)	1	1
Female	34.4 (24.8–45.3)	2.56 (1.59–4.14) ***	1.97 (1.04–3.72) *	17.6 (12.3–24.7)	0.50 (0.30–0.86) *	0.86 (0.40–1.83)	8.9 (5.9–13.4)	0.81 (0.53–1.25)	1.53 (0.80–2.95)
Age group (years)									
15–24	11.9 (7.3–19.0)	1	1	32.1 (25.8–39.1)	1	1	13.0 (8.5–19.3)	1	1
25–34	14.9 (10.0–21.7)	1.29 (0.82–2.05)	1.40 (0.60–3.31)	32.9 (27.0–39.3)	1.04 (0.72–1.49)	0.42 (0.19–0.91) *	12.5 (8.7–17.4)	0.95 (0.53–1.71)	0.95 (0.40–2.25)
35–44	17.5 (13.6–22.1)	1.56 (0.91–2.68)	1.72 (0.55–5.40)	29.2 (24.3–34.6)	0.87 (0.58–1.30)	0.60 (0.25–1.45)	14.6 (10.1–20.5)	1.14 (0.70–1.86)	1.36 (0.56–3.34)
45–54	20.5 (16.7–25.1)	1.91 (1.16–3.13) *	1.92 (0.76–4.84)	28.5 (19.7–39.3)	0.84 (0.47–1.51)	0.56 (0.25–1.28)	8.9 (5.3–14.6)	0.65 (0.33–1.28)	0.85 (0.35–2.05)
≥55	23.7 (18.2–30.3)	2.30 (1.33–3.96) **	2.75 (1.33–5.70) *	24.5 (18.1–32.3)	0.69 (0.40–1.18)	0.47 (0.20–1.09)	7.0 (5.1–9.7)	0.51 (0.36–0.71) ***	0.72 (0.27–1.93)
*P* trend		<0.001	<0.001		0.113	0.421		<0.001	0.161
Residence									
Urban	23.6 (14.7–35.6)	1	1	41.3 (23.1–62.2)	1	1	13.0 (8.0–20.4)	1	1
Rural	17.8 (14.6–21.6)	0.70 (0.37–1.35)	0.77 (0.39–1.52)	24.9 (21.6–28.5)	0.47 (0.18–1.26)	0.50 (0.19–1.37)	10.0 (7.1–13.9)	0.74 (0.39–1.43)	0.91 (0.37–2.20)
Ethnicity									
Lao	17.0 (11.6–24.2)	1		28.9 (21.1–38.2)	1		11.8 (8.0–17.3)	1	
Others ^a^	22.2 (17.3–28.1)	1.40 (0.83–2.36)		28.1 (23.5–33.1)	0.96 (0.58–1.60)		8.8 (6.3–12.1)	0.72 (0.39–1.30)	
Religion									
None	32.0 (16.1–53.6)	1		13.6 (7.7–23.0)	1		12.0 (3.3–35.6)	1	
Buddhist	17.9 (13.1–23.8)	0.46 (0.18–1.20)		30.4 (24.0–37.8)	2.77 (1.29–5.94) *		11.8 (8.4–16.3)	0.98 (0.22–4.34)	
Others ^b^	21.6 (15.7–28.9)	0.59 (0.27–1.26)		24.8 (20.0–30.4)	2.10 (0.99–4.45)		7.4 (5.4–10.1)	0.59 (0.17–2.04)	
Marital status									
Never married	11.4 (5.4–22.6)	1		34.5 (26.1–43.9)	1		18.9 (12.5–27.5)	1	
Currently married	19.7 (15.9–24.1)	1.91 (1.00–3.65)		28.1 (23.2–33.5)	0.74 (0.50–1.10)		10.2 (7.5–13.8)	0.49 (0.31–0.79) **	
Divorced/ Separated/ Widowed	22.5 (15.4–31.6)	2.25 (1.08–4.68) *		27.4 (16.7–41.5)	0.72 (0.41–1.25)		5.2 (2.2–11.5)	0.24 (0.07–0.81) *	
Education level									
Never attended school	24.7 (17.9–33.1)	1	1	14.5 (10.9–19.0)	1	1	7.3 (3.8–13.6)	1	1
Primary school	17.5 (14.0–21.8)	0.65 (0.43–0.99) *	0.93 (0.58–1.50)	26.1 (23.1–29.2)	2.09 (1.51–2.88) ***	2.40 (1.44–4.01) **	9.0 (7.0–11.6)	1.26 (0.63–2.50)	1.28 (0.55–2.94)
Secondary school	15.9 (11.5–21.6)	0.58 (0.34–0.98) *	1.05 (0.60–1.83)	32.7 (26.5–39.6)	2.88 (1.88–4.41) ***	2.53 (1.38–4.64) **	13.0 (8.5–19.2)	1.90 (0.84–4.27)	2.80 (1.37–5.72) **
High school or higher	23.0 (15.7–32.4)	0.91 (0.50–1.67)	1.26 (0.75–2.12)	47.9 (33.2–62.9)	5.44 (2.96–9.99) ***	4.82 (2.14–10.87) **	19.6 (13.0–28.3)	3.09 (1.27–7.52) *	3.18 (1.21–8.31) *
*P* trend		0.702	0.272		<0.001	0.008		0.008	<0.001
Average income per household member per day in US dollars									
<1.9 ^c^	17.6 (14.2–21.7)	1	1	25.2 (21.7–28.9)	1	1	10.1 (7.5–13.5)	1	1
≥1.9	20.8 (15.1–28.0)	1.23 (0.84–1.79)	1.17 (0.85–1.60)	32.8 (23.6–43.5)	1.45 (1.03–2.04)	1.21 (0.98–1.49)	13.3 (7.1–23.4)	1.36 (0.67–2.78)	1.26 (0.64–2.50)
Occupation									
Unemployed	21.6 (13.1–33.5)	1		21.9 (13.8–32.9)	1		3.8 (1.5–9.1)	1	
Government sector	24.7 (14.8–38.2)	1.19 (0.83–1.71)		42.7 (33.5–52.3)	2.66 (1.64–4.32) ***		19.7 (13.1–28.5)	6.27 (2.28–17.25) **	
Non-government company/organization	22.2 (16.6–29.0)	1.03 (0.65–1.66)		21.1 (10.4–38.1)	0.96 (0.61–1.50)		4.3 (1.3–12.9)	1.15 (0.35–3.72)	
Agriculture	17.1 (14.0–20.7)	0.75 (0.44–1.29)		28.3 (25.4–31.4)	1.41 (0.80–2.50)		11.4 (8.1–15.9)	3.30 (1.11–9.81) *	
Non-farm self-employed	17.1 (11.6–24.5)	0.75 (0.40–1.42)		38.6 (27.1–51.6)	2.25 (1.35–3.75) **		20.7 (10.9–35.7)	6.67 (2.17–20.49) **	
Others	27.2 (19.6–36.4)	1.36 (0.79–2.34)		32.5 (18.2–51.1)	1.72 (1.05–2.84) *		12.2 (5.7–24.3)	3.56 (1.01–12.54) *	
Smoking frequency									
Daily	19.3 (15.4–23.8)	1	1	26.1 (21.1–31.8)	1	1	9.8 (7.3–13.0)	1	1
Occasional (less than daily)	17.1 (13.0–22.3)	2.98 (1.79–4.96) ***	3.08 (1.73–5.48) *	61.5 (50.7–71.3)	4.52 (2.66–7.68) ***	3.77 (2.19–6.50) *	31.2 (18.9–46.8)	4.17 (2.11–8.23) ***	5.51 (2.77–10.93) *
Number of cigarettes smoked per day, median (Q1–Q3)				10 (5–16)	0.97 (0.94–0.99) **		10 (5–15)	0.95 (0.93–0.97) ***	
Time to smoking the first cigarette of the day after waking									
≤30 min	6.3 (4.2–9.5)	1		25.4 (18.3–34.2)	1		9.3 (5.9–14.3)	1	
>30 min	7.5 (4.4–12.7)	1.21 (0.75–1.94)		26.7 (22.1–31.8)	1.07 (0.75–1.52)		10.2 (7.9–13.0)	1.11 (0.73–1.68)	
Visited a health-care provider during the past 12 months									
No	15.9 (13.0–19.3)	1	1	25.9 (20.7–31.9)	1	1	10.6 (7.8–14.3)	1	1
Yes	30.7 (22.7–40.0)	2.34 (1.70–3.22) ***	1.86 (1.23–2.80) **	39.6 (33.1–46.5)	1.87 (1.37–2.56) ***	1.74 (1.28–2.35) ***	10.3 (6.6–15.8)	0.97 (0.58–1.64)	1.07 (0.63–1.81)
Ever received advice to quit smoking from health care providers									
No	7.6 (4.1–13.9)	1	1	36.9 (30.7–43.5)	1	1	10.6 (6.5–16.6)	1	1
Yes	9.8 (2.7–29.8)	1.32 (0.53–3.29)	0.80 (0.41–1.55)	51.3 (39.4–63.0)	1.81 (1.22–2.67) **	1.67 (0.90–3.12)	9.1 (2.6–27.2)	0.85 (0.21–3.34)	0.60 (0.12–2.92)
Exposed to secondhand smoking at indoor workplace in the past 30 days									
No	31.2 (20.1–44.9)	1	1	47.3 (28.5–66.8)	1	1	35.5 (23.8–49.2)	1	1
Yes	15.7 (10.7–22.5)	0.41 (0.20–0.84) *	0.33 (0.15–0.71) **	33.3 (26.1–41.3)	0.56 (0.20–1.54)	0.45 (0.07–2.95)	7.1 (3.9–12.7)	0.14 (0.05–0.40) **	0.33 (0.06–1.69)
Exposed to secondhand smoking at indoor public places (including buildings, healthcare facilities, restaurants, food store, and public transportation vehicles) in the past 30 days									
No	18.6 (14.2–24.0)	1	1	27.9 (23.2–33.2)	1	1	9.1 (6.2–13.2)	1	1
Yes	20.4 (16.4–25.1)	1.12 (0.85–1.48)	1.35 (0.93–1.95)	29.7 (23.5–36.7)	1.09 (0.87–1.37)	0.90 (0.69–1.17)	13.3 (9.7–17.9)	1.53 (0.95–2.45)	1.34 (0.79–2.27)
Smoking rule at home									
Allowed	17.5 (14.5–21.0)	1	1	27.5 (23.3–32.1)	1	1	10.6 (8.0–14.0)	1	1
Not allowed	55.2 (49.2–61.1)	5.80 (4.31–7.80) ***	4.86 (3.42–6.92) ***	78.5 (68.6–85.9)	9.62 (5.22–17.73) ***	5.52 (2.13–14.33) ***	0		
Noticed media-based messages informing the dangers of smoking or encouraging quitting in the past 30 days									
No	18.2 (14.5–22.6)	1	1	13.5 (9.3–19.4)	1	1	5.7 (3.1–10.3)	1	1
Yes	19.8 (15.2–25.4)	1.11 (0.82–1.50)	1.00 (0.72–1.40)	36.8 (31.6–42.3)	3.72 (2.81–4.92) ***	3.25 (2.28–4.63) ***	14.2 (10.6–18.8)	2.75 (1.41–5.38) **	2.83 (1.34–5.96) **
Noticed health warnings on cigarette packages in the past 30 days									
No				16.3 (12.0–21.7)	1	1	2.0 (1.0–3.8)	1	1
Yes				33.9 (28.0–40.3)	2.64 (1.81–3.84) ***	3.33 (2.21–5.03) ***	14.3 (10.6–19.0)	8.18 (3.99–16.79) ***	8.31 (2.88–23.96) ***
Believed that smoking is harmful to participants’ health									
No, not harmful	30.8 (22.0–41.2)	1	1	14.8 (7.2–28.0)	1	1	5.7 (1.9–15.7)	1	1
Yes, a little harmful or moderately harmful	12.2 (9.5–15.6)	0.31 (0.22–0.45) ***	1.25 (0.66–2.36)	16.6 (9.3–27.7)	1.14 (0.50–2.61)	1.48 (0.42–5.29)	6.4 (3.1–12.6)	1.13 (0.27–4.76)	0.61 (0.11–3.50)
Yes, seriously harmful	21.6 (17.2–26.9)	0.62 (0.37–1.04)	1.82 (0.84–3.95)	36.7 (32.7–41.0)	3.34 (1.50–7.45) **	3.45 (1.24–9.57) *	14.6 (10.3–20.3)	2.86 (0.84–9.68)	3.22 (0.64–16.19)
*P* trend		0.114	0.001		0.001	0.001		0.027	0.003
Believed that smoking causes illnesses (including bronchitis, lung cancer, or heart diseases)									
No	12.9 (8.9–18.4)	1	1	23.1 (16.8–30.7)	1	1	2.2 (0.7–6.5)	1	1
Yes	21.9 (17.7–26.8)	1.89 (1.29–2.77) **	1.79 (1.04–3.08) *	29.1 (22.5–36.7)	1.37 (0.87–2.14)	1.30 (0.72–2.35)	10.7 (6.6–16.7)	5.36 (1.38–10.78) *	5.06 (1.29–14.97) *
Believed that it is a sin for a cigarette or pipe smoker to produce smoke that is inhaled by other persons									
No	16.1 (10.0–24.9)	1	1	30.6 (23.6–38.6)	1	1	6.9 (4.1–11.6)	1	1
Yes	17.5 (13.8–22.1)	1.11 (0.75–1.65)	1.33 (0.76–2.33)	30.6 (25.5–36.2)	1.00 (0.82–1.23)	1.00 (0.72–1.38)	13.0 (9.8–17.1)	2.01 (1.06–3.82) *	2.04 (0.82–5.09)
Believed that “A man who does not smoke is not a real man”									
No	19.2 (14.9–24.2)	1	1	30.3 (25.6–35.5)	1	1	13.4 (9.7–18.1)	1	1
Yes	14.0 (10.4–18.6)	0.69 (0.54–0.87) **	0.63 (0.47–0.85) **	30.6 (21.8–41.0)	1.01 (0.72–1.43)	0.84 (0.51–1.38)	6.0 (2.6–13.0)	0.41 (0.16–1.06)	0.34 (0.12–0.96) *
Believed that the price of cigarettes should be raised to encourage people to stop smoking									
No	12.9 (10.8–15.3)	1	1	24.0 (19.0–29.9)	1	1	8.1 (6.1–10.7)	1	1
Yes	23.4 (18.1–29.6)	2.06 (1.55–2.73) **	2.04 (1.57–2.64) **	36.3 (30.9–42.1)	1.80 (1.41–2.30) **	1.17 (0.79–1.73)	16.0 (11.7–21.6)	2.16 (1.67–2.80) **	2.24 (1.36–3.68) **

^a^ including PhouThai, Khermou, Khamu, Khmu, Leu, Mong, etc. ^b^ including Christian, Pee, Phi, Phy, Pi, etc. ^c^ International poverty line (http://povertydata.worldbank.org/poverty/country/LAO) ^d^ Adjusted for sex, age groups, education level, income per day, and smoking frequency. * *p* < 0.05, ** *p* < 0.01, *** *p* < 0.001.
